# Differential human urinary lipid profiles using various lipid-extraction protocols: MALDI-TOF and LIFT-TOF/TOF analyses

**DOI:** 10.1038/srep33756

**Published:** 2016-09-20

**Authors:** Phornpimon Tipthara, Visith Thongboonkerd

**Affiliations:** 1 Medical Proteomics Unit, Office for Research and Development, Faculty of Medicine Siriraj Hospital; and Center for Research in Complex Systems Science (CRCSS), Mahidol University, Bangkok, Thailand

## Abstract

Changes in lipid levels/profiles can reflect health status and diseases. Urinary lipidomics, thus, has a great potential in clinical diagnostics/prognostics. Previously, only chloroform and methanol were used for extracting lipids from the urine. The present study aimed to optimize lipid extraction and examine differential lipid classes obtained by various extraction protocols. Urine samples were collected from eight healthy individuals and then pooled. Lipids were extracted by six solvent protocols, including (i) chloroform/methanol (1:1, v/v), (ii) chloroform/methanol (2:1, v/v), (iii) hexane/isopropanol (3:2, v/v), (iv) chloroform, (v) diethyl ether, and (vi) hexane. Lipid profiles of the six extracts were acquired by MALDI-TOF mass spectrometry (MS) and some lipid classes were verified by LIFT-TOF/TOF MS/MS. The data revealed that phosphatidylglycerol (PG) and phosphatidylinositol (PI) could be detected by all six protocols. However, phosphatidylcholine (PC) and sphingomyelin (SM) were detectable only by protocols (i)–(iv), whereas phosphatidylserine (PS) was detectable only by protocols (iii)–(vi), and phosphatidylethanolamine (PE) was detectable only by protocols (v)–(vi). In summary, we have demonstrated differential lipidome profiles yielded by different extraction protocols. These data can serve as an important source for selection of an appropriate extraction method for further highly focused studies on particular lipid classes in the human urine.

Urine is a versatile sample widely used for clinical diagnostics as its collection from patients or healthy subjects is very simple and noninvasive^1^. More importantly, it is accessible and available in almost all patients. Human urine normally contains very tiny amount of lipids, of which levels are increased in some kidney diseases. Lipiduria is found in urine of patients with nephrotic syndrome, in which levels of cholesterol, cholesterol ester, triacylglycerides, free fatty acid, phosphatidylcholine (PC), phosphatidylethanolamine (PE) and phosphatidylserine (PS) are elevated and can be used as the diagnostic markers^2^. Difference of sphingolipid (SP) profile in urine of patient with Fabry disease can be used for discriminating type of Fabry disease^3^. Additionally, PE and PS are detected in the urine of patients with metabolic disorder caused by dysfunction of mitochondrial DNA^4^. Moreover, phospholipids (PLs), which are the highly abundant component of renal epithelial cell membrane, are detectable in the urine of kidney stone formers^5^,^6^. There is a study showing that PLs play a significant role in crystal nucleation and kidney stone formation^7^. Several animal studies have demonstrated that phospholipiduria is a result of damaged renal epithelial cell membranes after animals are exposed to certain drugs or toxic chemicals^8^. Thus, urinary lipid profiling has a great potential in clinical diagnostics and prognostics.

Unlike proteins or genes, which are made up of a limited number of monomeric units, lipids exhibit a much greater degree of molecular structural diversity, resulting to technical challenges for their analysis. Traditional lipid analysis, i.e. thin layer chromatography (TLC), is limited by technical sensitivity, selectivity and resolution^9^,^10^. Moreover, TLC procedures are time-consuming^9^,^10^. The emerging field of lipid analytical technology using mass spectrometry has enabled the analysis of complex lipid mixtures more feasible and revealed the important roles of lipids in many biological cell processes, particularly cell membrane structure and energy storage^9^,^10^. Gas chromatography coupled with mass spectrometry (GC-MS), however, has a disadvantage from its detection limit^9^,^10^. Only volatile lipids, i.e. volatile fatty acids, can be detected^9^,^10^. Moreover, derivatization step prior to analysis needs further improvement. The development of soft ionization techniques, including electrospray ionization (ESI)^11^ and matrix-assisted laser desorption/ionization (MALDI)^12^, may expand the range of lipids that can be detected.

Sample preparation and processing prior to analysis is a crucial step for lipid profiling. Previously, urinary lipid extraction was mostly done by using the solvent mixture of chloroform and methanol (1:1 or 2:1, v/v)^2^,^3^,^5^,^7^,^13^. Indeed, lipids can be extracted by other solvents. However, there was no previous study that used extraction method other than chloroform/methanol to extract lipids from human urine. The present study thus aimed to characterize human urinary lipidome by MALDI-TOF/TOF mass spectrometry^14^,^15^ and to compare urinary lipidome profiles recovered by various extraction protocols.

## Results and Discussion

**Figure 1:**
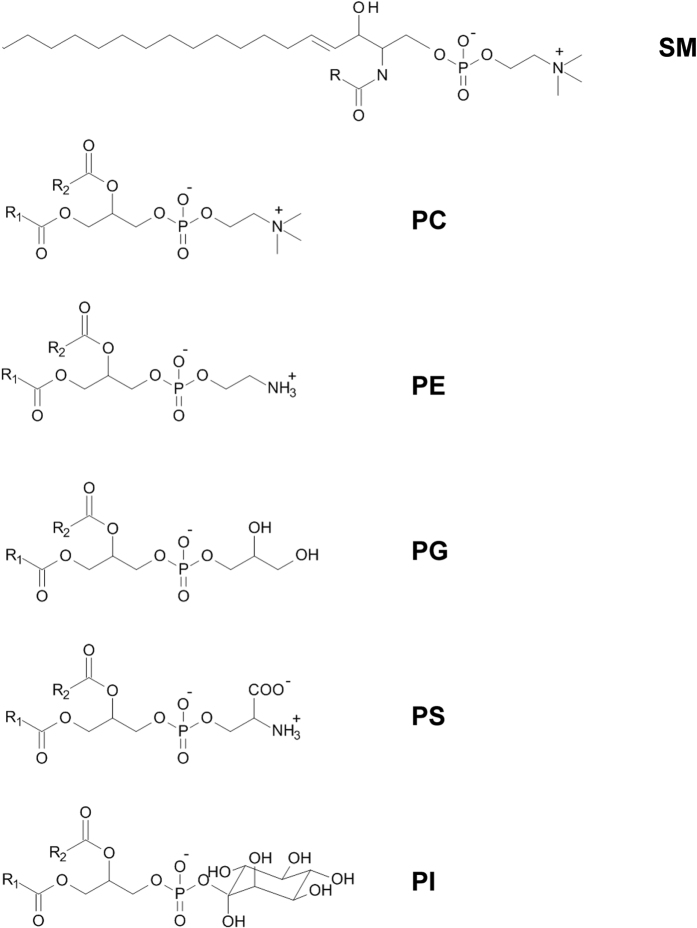
Chemical structures of most common lipid classes excreted into the urine. Sphingomyelin (SM) and phosphatidylcholine (PC) containing trimethyamine group (−CH_2_CH_2_N(CH_3_)_3_) are the most abundant compositions and can be detected as the protonated ([M + H]^+^) and and sodiated ([M + Na]^+^) ions.

The present study examined normal human urinary lipidome profile by MALDI-TOF MS and LIFT-TOF/TOF MS/MS analyses using various lipid extraction protocols (Table 1). The most relevant lipids in the urine, of which chemical structures are shown in Fig. 1, could be detected by MALDI-TOF MS profiling. Among these, sphingomyelin (SM), which is a class of sphingolipids (SPs), phosphatidylcholine (PC), phosphatidylserine (PS), and phosphatidylethanolamine (PE) are the main components, whereas phosphatidylglycerol (PG) and phosphatidylinositol (PI) are less abundant constituents of the membrane structures. It would be expected that PG and PI might be more difficult to be detected by conventional lipid extraction protocols.

**Figure 2:**
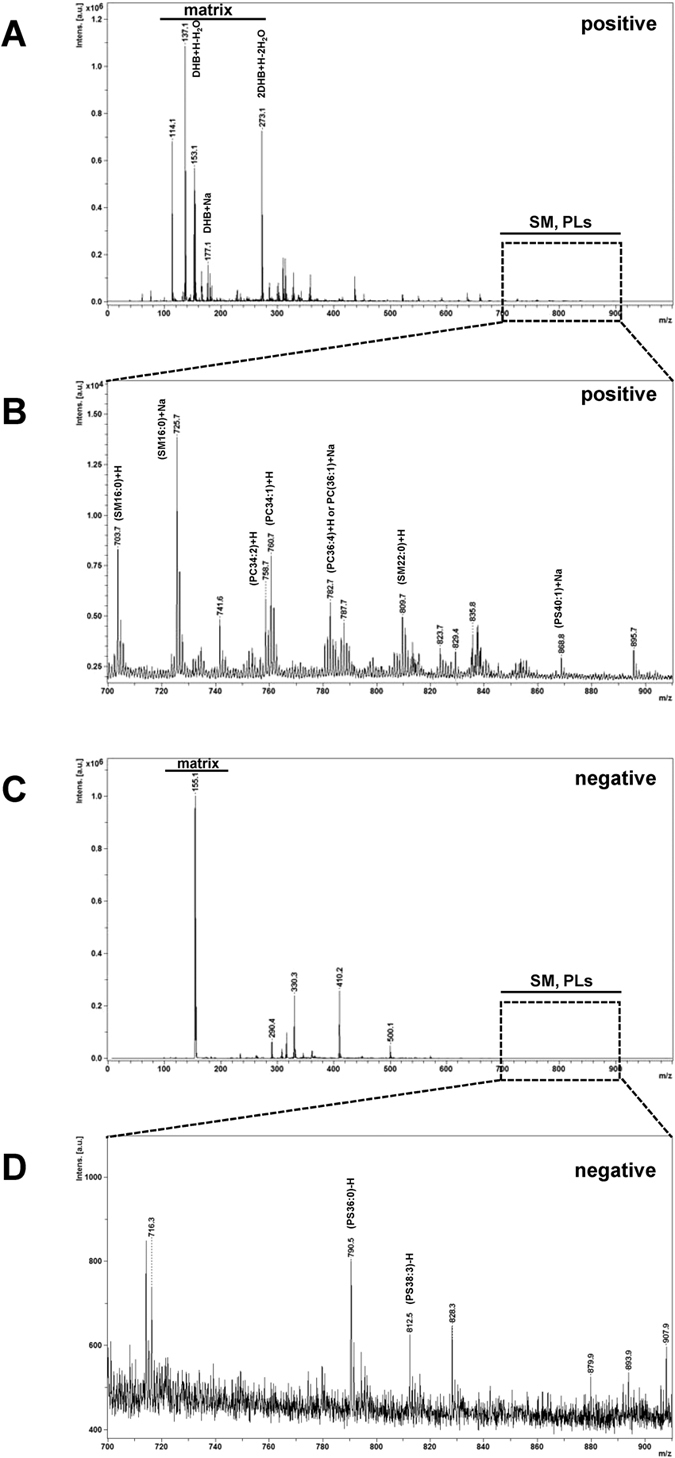
Example of MALDI-TOF MS spectra of lipid extracted from the pooled urine using protocol (i) (C:M_1:1). (**A,C)** MS spectra were acquired by positive and negative ionization modes, respectively, in the *m/z* range of 0–1,000. DHB matrix ions were detected as three ion forms in the low-mass range. **(B,D)** Zoom-in MS spectra in the *m/z* range of 700–910, which covered the intact mass range of almost all of phospholipids (PLs) and sphingomyelin (SM), which is a class of sphingolipids (SPs).

Due to the chemical diversity of lipids, their analysis in this study was performed in both positive and negative ionization modes. Fortunately, DHB matrix could be used for lipid analysis in positive and negative ionization modes as previously described^16^. With the advantage of MALDI-TOF that sample on the MALDI plate from previous acquisition can be re-acquired, we thus acquired the lipid spectra from the positive ion mode followed by the negative ion mode on the same sample spots. Examples of MALDI-TOF MS spectra of lipids derived from the pooled urine using protocol (i) (C:M_1:1) and detected by positive and negative ionization modes are illustrated in Fig. 2. The matrix molecule, 2,5-dihydroxybenzoic acid (DHB), was detected as three adduct ions, including [M + H-H_2_O]^+^ (*m/z* = 137.1), [M + Na]^+^ (*m/z* = 177.1), and [2M + H-2H_2_O]^+^ (*m/z* = 273.1). These DHB adduct ions were consistent with the previous studies using 0.5 M DHB in methanol with 0.1% TFA as the matrix solution^17^, and thus were used to normalize the abundance of each lipid species identified in this study. Fig. 2B,D show the zoom-in spectra obtained from positive and negative ionization modes, respectively, in the *m/z* range of 700–910, which cover the intact masses of almost all of phospholipids (PLs) and the SM class of SPs.

It was obvious that the positive ionization mode covered a greater number of the lipid species as compared to that acquired by negative ionization mode, consistent with the data reported previously^16^. This is because most of the lipid species can be ionized by positive ionization mode easier than by using the negative mode^12^. Moreover, the data acquired from the negative ionization mode showed no significant differences among the six extraction protocols. Therefore, subsequent analyses focused mainly on MALDI-TOF MS and MS/MS spectra of the lipid extracts acquired by positive ionization mode.

**Figure 3:**
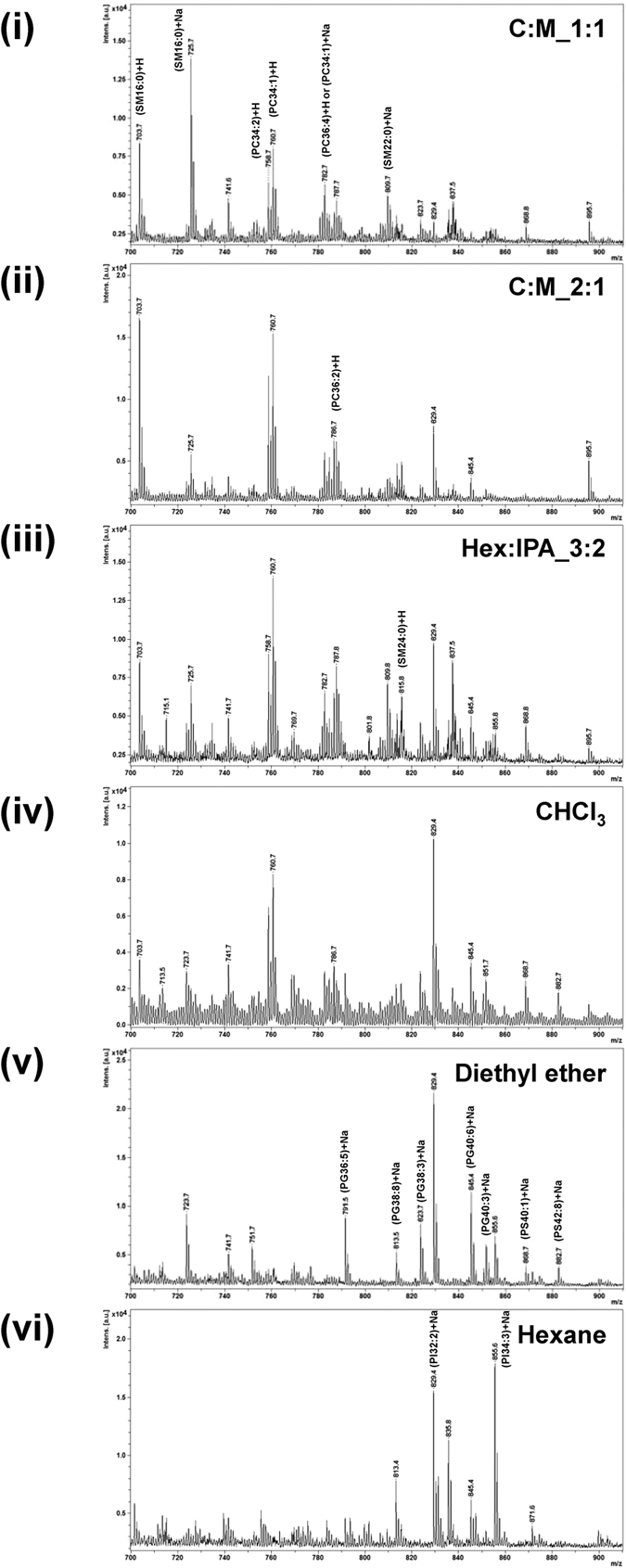
Comparison of urinary lipid profile acquired by positive ionization mode in the *m/z* range of 700–910 of the extracts recovered from six different extraction protocols.

Figure 3 shows zoom-in MS spectra (*m/z* = 700–910) of lipid extracts from individual solvent protocols acquired by positive ionization mode. A comparison of six extraction protocols revealed markedly different lipid profiles. The assigned lipid classes, including SM, PC, PE, PG, PI and PS, and their detectability (in triplicate set of experiments) by each extraction protocol are summarized in Table 2. The details of their chemical structures of these lipid species indicating fatty acid composition identified from LIPID MAPS Structure Database are shown in Supplementary Table S1. Moreover, the inter-assay consistency of the data derived from different experiments was achieved and is shown in Supplementary Figures S1 and S2 to strengthen the validity of our extraction protocols and existing differential lipid profiles recovered from such various protocols.

From Table 2, SM16:0 and SM22:0 were identified only in the extracts of protocols (i)–(iv), but could not be identified from protocols (v) and (vi). SM24:0 was detectable only in the extracts of protocols (i)–(iii). Similar to SM16:0 and SM22:0, PC34:2, PC34:1, PC36:4, and PC36:2 were identified in the extracts of protocols (i)–(iv), not in the extracts derived from protocols (v) and (vi). In contrast, PE42:2 was identified only in protocols (v) and (vi), but could not be detected by protocols (i)–(iv). Interestingly, PS species were identified in protocols (iii)–(vi), not in (i) and (ii). Moreover, PG and PI species were detected in all the six extraction protocols (Table 2).

Interestingly, the spectrum at *m/z* of 855.6 corresponded to [M + Na]^+^ion of PI34:3. It was enriched in extracts from protocols (v) and (vi), while could be also detected in protocol (iii) (Table 2 and Fig. 3). Additionally, the spectral intensity of *m/z* 829.4, which corresponded to [M + Na]^+^ion of PI32:2, was gradually increased from protocols (i) to (vi) (Fig. 3). PI is a phospholipid that plays an important role in regulation of a wide variety of cellular processes. Previously, detection of PI species from the brain lipid mixture using MALDI-MS could be done only by prior elimination of PC^18^. Therefore, our finding suggested that in the study of specific PI species using MALDI-TOF MS, nonpolar solvent, such as hexane, is more suitable than polar solvents for extracting PI from a complex lipid mixture.

Similar to PI, PG36:5, PG38:8, PG38:3, PG40:6 and PG40:3 were detected by all protocols, but with a gradual increase in their intensities from protocols (i) to (vi) (Fig. 3). For PS, PS40:1 and PS42:8 were detected only in the extracts from protocols (iii)–(vi). Interestingly, the PI, PG, and PS species identified in our present study had not been previously identified in human urine by mass spectrometry^19^. This indicated that our lipidome profiling using various protocols could enhance the lipid profiling in the human urine. A hydrophilic head group of lipid is important for selective extraction by various solvents. Low-polarity solvents, i.e. diethyl ether and hexane in protocols (v) and (vi), respectively, could not extract PC and SM, which contain a hydrophilic choline head group. On the other hand, polar alcoholic mixture solvents in protocols (i)–(iii) and intermediate polarity solvent, i.e. chloroform in protocol (iv), offered high recovery yield of PC and SM extraction (Table 2).

In addition to lipid identification, we quantitated the relative abundance of each lipid class obtained from individual extraction protocols. We observed that profiling of the 2,5-dihydroxybenzoic acid (DHB) adduct ions acquired in positive ion mode was reproducible in all extraction protocols (with total of 18 mass spectra from triplicate set of six protocols analyzed). Among these DHB adducts, ratio of the peak intensity of *m/z* 137.0 to *m/z* 273.1 (intensity of *m/z* 137.0 divided by intensity of *m/z* 273.1) had the lowest coefficient of variation (CV) at 5%. We therefore used this ratio to normalize the intensity of all the lipid species acquired throughout the present study.

**Figure 4:**
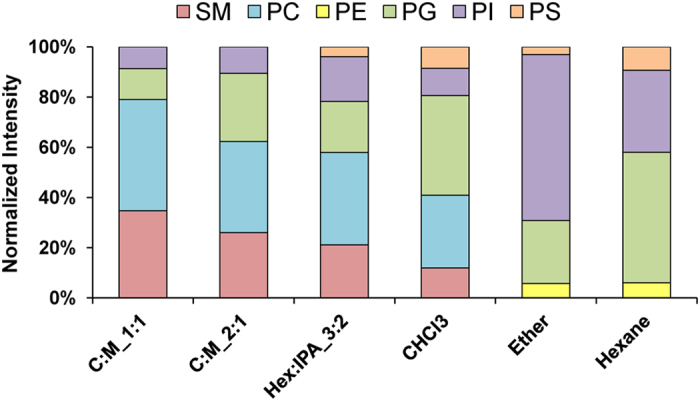
Quantitative analysis of individual urinary lipid classes recovered by using six different lipid extraction protocols. The spectral intensity of each lipid species was normalized with intensity ratio of the two matrix spectra at *m/z* of 137.0 and 273.1, which gave the lowest coefficient of variation (CV) at 5%. The abundance of all the lipid classes identified by each protocol is presented as percentage of the total lipids recovered.

Figure 4 summarizes the quantitative data of relative abundance of each lipid class obtained from individual extraction protocols. The data showed that there was no any single extraction protocol that could yield all the lipid classes from the human urine. This data was in accordance with the limitation of human urinary proteomics (and proteomic analysis of other biological samples) in which one sample preparation protocol cannot yield the entire proteome profile in a given sample^20^. Combination of more than one method may be required to achieve the whole lipidome/proteome profile of the human urine^20^. The data also showed that PG and PI could be detected by all six protocols. As PG and PI are the low abundant lipid species as compared to other species, this data suggested that our extraction protocol is quite successful to enrich and recover these two lipid species from the human urine and will be useful in clinical applications. However, PC and SM were detectable only by protocols (i)–(iv), whereas PS was detectable only by protocols (iii)–(vi), and PE was detectable only in protocols (v) and (vi). The latter sets of the data indicated the selectivity of each extraction protocol that can be selected for suitable clinical applications.

**Figure 5:**
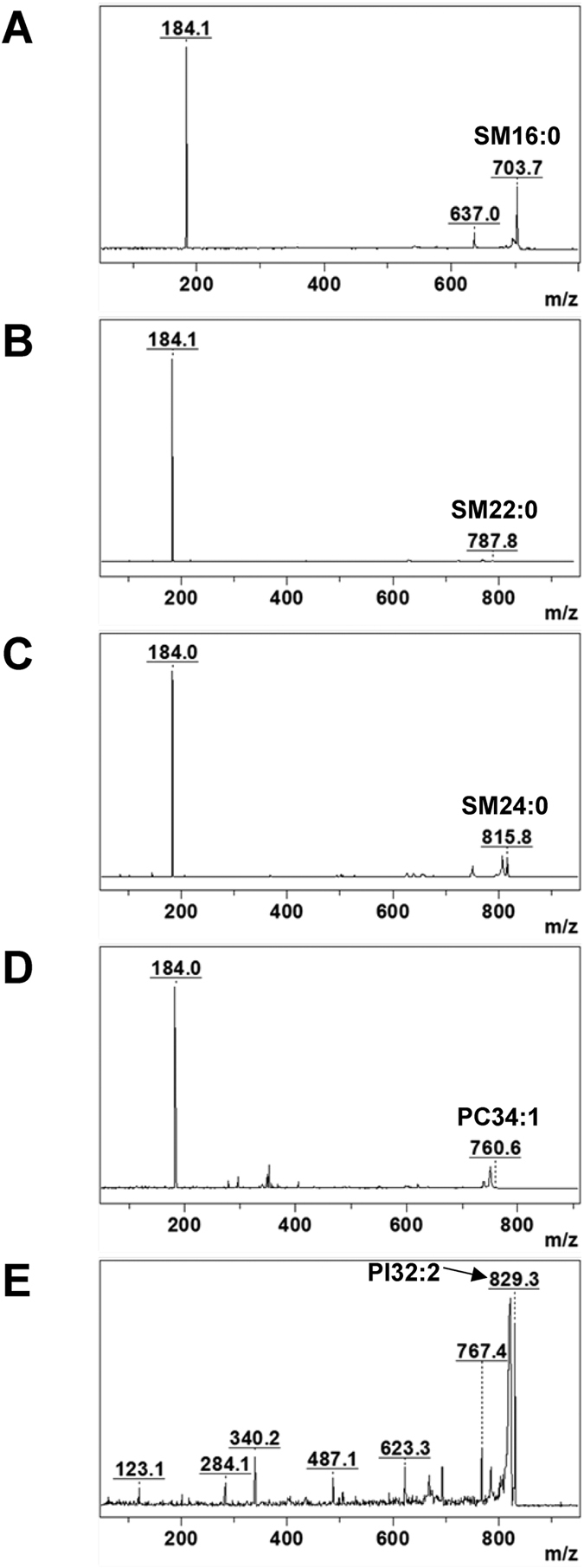
Confirmation of some major abundant lipid classes by LIFT-TOF/TOF analysis. MS/MS spectra were acquired using positive ionization mode. (**A**) [M + H]^+^ of SM16:0. (**B)** [M + H]^+^ of SM22:0. (**C**) [M + H]^+^ of SM24:0. (**D**) [M + H]^+^ of PC34:1. (**E)** [M + Na]^+^ of PI32:2.

Finally, tandem mass spectrometry (MS/MS) was performed on some major abundant lipid species using LIFT-TOF/TOF mode. The product ions characteristic head-group of the lipid was used to identify the lipid class. Fragment ions were generated by LIFT technology as described previously^15^. Figure 5 shows examples of MS/MS spectra acquired by LIFT TOF/TOF mode to confirm SM, PC and PI lipid classes. The results showed that SMs and PCs could be readily identified by the characteristic *m/z* of 184 head group fragment ion, whereas PIs could be identified by the characteristic *m/z* of 284 fragment ion.

Investigations of SM and PC are quite important as they are relevant to many diseases. In our present study, detection of these two lipid classes by MALDI-TOF MS and LIFT-TOF/TOF MS/MS analysis was relatively simpler as compared to other lipid classes due to the high abundant levels of SM and PC in the urine. In concordance, there are previous reports indicating the high abundance of PC and SM in human plasma^21^, and in HDL and LDL using MALDI-TOF MS^17^. The detection of lipids by MALDI-TOF MS depends not only on the chemical composition and structure of the target molecule, which determines its inherent ionizability, but also on the presence of other compounds in the complex sample or extract, which can compete for charges or suppress signals through gas-phase reactions. Thus, the selective extraction or enrichment of lipids of interest (or removal of the unwanted but predominant components) can improve the detection of low abundant lipids^18^. Our data suggest that diethyl ether and hexane may be the solvents of choice to extract low abundant lipids such as PI, PE and PG in lipid mixtures that contain high abundant SMs and PCs. As there are no detectable spectra of SM or PC in these extracts, protocols (v) and (vi) do not require prior separation/elimination of SM and PC, making them feasible in clinical applications.

In summary, we have successfully established methods for selective extraction of differential lipid components in the human urine. Our results showed that PG and PI could be detected by all six protocols. However, PC and SM were detectable only by protocols (i)–(iv), whereas PS was detectable only by protocols (iii)–(vi), and PE was detectable only by protocols (v) and (vi). These data can serve as an important source for selection of an appropriate extraction method for further highly focused studies on particular lipid classes in the human urine.

## Materials and Methods

### Chemicals

Methanol, hexane, and isopropanol were HPLC-grade from Fisher Scientific (Loughborough, UK). Chloroform was HPLC-grade from RCI Labscan (Bangkok, Thailand). Diethyl ether was from Merck (Darmstadt, Germany). Trifluoroacetic acid (TFA) was MS-grade from Fisher Scientific (Loughborough, UK). 2,5-dihydroxybenzoic acid (DHB) was from Bruker Daltonik (Bremen, Germany). Deionized water (18.2 MΩ·cm) was derived from Milli-Q water purification system (Millipore; Bedford, MA).

### Urine collection

This study was approved by the institutional ethical committee (Siriraj Institutional Review Board) (approval no. Si650/2015). All the experiments involved human subjects and clinical samples were conducted according to the international guidelines, i.e. the Declaration of Helsinki, the Belmont Report, and ICH Good Clinical Practice, and informed consent was obtained from all subjects. Mid-stream urine samples were collected from 8 healthy individuals (4 males and 4 females). An equal volume from each urine sample was pooled and then centrifuged at 1,000 *g* and room temperature (RT; set at 25 °C) for 5 min to remove cellular debris and particles. The clear supernatant was collected and processed for lipid extraction.

### Lipid extraction

Extraction of lipids from the pooled urine was performed by using 6 different solvent protocols (summarized in Table 1), each was performed in triplicate. A total of 6 ml of solvent from each protocol was added to 2 ml of urine and incubated at RT for 15 min. Separation of the organic solution into two phases was achieved by adding 1 ml of deionized water, vortexed for 1 min, and centrifuged at 1000 *g* and 20 °C for 5 min. The organic phase layer (containing lipids) was then collected using a pipette and transferred into a new tube. The extract was then dried in a SpeedVac concentrator (Savant; Holbrook, NY).

### Mass spectrometry

MALDI-TOF MS was performed using an UltrafleXtreme MALDI-TOF/TOF mass spectrometer (Bruker Daltonik; Bremen, Germany). The TOF was calibrated using a peptide mixture (Bruker Daltonik) before acquisition of the sample spectra. The dried lipid extracts were reconstituted in 200 μl of CHCl_3_/MeOH (2:1, v/v). Equal volume (1–2 μl) of lipid extract and MALDI matrix solution (0.5 M 2,5-dihydroxybenzoic acid, DHB, in methanol containing 0.1% trifluoroacetic acid) was mixed in a 0.6-ml microcentrifuge tube. Then, 1 μl of the mixture was spotted onto a MALDI plate (Bruker Daltonik). The mass spectra were acquired over the *m/z* range of 0–1,000 in both positive and negative ion Reflectron TOF modes. The laser power was adjusted to a point just above the ionization threshold of the sample, and the laser rate was set at 10 Hz with 1,000 laser shots per acquisition. Ten acquisitions were averaged for each individual sample.

Tandem mass spectrometry (MS/MS) analysis was performed on molecular species of interest (some major abundant lipid classes) using LIFT-TOF/TOF mode. The product ions characteristic head-group of the lipid was used to identify the lipid class. Fragment ions were generated by LIFT (laser ionization fragmentation technology) approach as described previously^15^.

### Data analysis

A mass list was generated by peak detection algorithm within FlexAnalysis software (Bruker Daltonik). Signal to noise threshold of 4, peak width of 0.1 *m/z*, and Tophat base line subtraction were used as the default parameters. The *m/z* values of lipid ions were searched against LIPID MAPS Structure Database (LMSD)^22^ using the LIPID MAPS tool (www.lipidmaps.org/tools)^23^. The [M + H]^+^ and [M + Na]^+^ were selected in the positive ionization mode, whereas [M−H]^−^ was selected in the negative ionization mode. The mass tolerance was set at ±0.2 Da. Lipid categories were limited to phospholipids (PLs) and sphingolipids (SPs). The relative intensity of each lipid class identified was normalized by intensity ratio of the two matrix ions, *m/z* of 137.0 and 273.1 (intensity of *m/z* 137.0 divided by intensity of *m/z* 273.1), which gave the lowest coefficient of variation (CV) among all the DHB adduct ions.

## Additional Information

**How to cite this article**: Tipthara, P. and Thongboonkerd, V. Differential human urinary lipid profiles using various lipid-extraction protocols: MALDI-TOF and LIFT-TOF/TOF analyses. *Sci. Rep*. **6**, 33756; doi: 10.1038/srep33756 (2016).

## Supplementary Material

Supplementary Information

## Figures and Tables

**Table 1:** Six solvent systems used for individual extraction protocols.

Protocol	Description	Solvent(s) used
(i)	C:M_1:1	chloroform/methanol (1:1, v/v)
(ii)	C:M_2:1	chloroform/methanol (2:1, v/v)
(iii)	Hex:IPA_3:2	hexane/isopropanol (3:2, v/v)
(iv)	CHCl_3_	chloroform
(v)	Ether	diethyl ether
(vi)	Hexane	hexane

**Table 2:** Summary of lipid species identified from the human urine using different extraction protocols.

Lipid species	*m/z*	Adduct ion	Protocol (i) C:M_1:1	Protocol (ii) C:M_2:1	Protocol (iii) Hex:IPA_3:2	Protocol (iv) CHCl_3_	Protocol (v) Ether	Protocol (vi) Hexane
SM16:0	703.7/725.7	[M + H]^+^/[M + Na]^+^	+++	+++	+++	++	0	0
SM22:0	787.8/809.7	[M + H]^+^/[M + Na]^+^	+++	++	+++	+	0	0
SM24:0	815.8	[M + H]^+^	+	++	+++	0	0	0
PC34:2	758.7	[M + H]^+^	++ +	+++	+++	+++	0	0
PC34:1	760.6	[M + H]^+^	+++	+++	+++	+++	0	0
PC36:4	782.6	[M + H]^+^	+++	+++	+++	+++	0	0
PC36:2	786.7	[M + H]^+^	+++	++	++ +	++	0	0
PE42:2	850.7	[M + H]^+^	0	0	0	0	+	+++
PG36:5	791.5	[M + Na]^+^	0	+	+	++	+++	+
PG38:8	813.4	[M + Na]^+^	+	+	+	+++	+++	+++
PG38:3	823.7	[M+Na]^+^	0	+	++	+++	+++	+
PG40:6	845.4	[M+Na]^+^	0	+++	+++	+	+++	+++
PG40:3	851.7	[M+Na]^+^	0	+	+	+	+++	++
PI32:2	829.4	[M+Na]^+^	+	+++	+++	+++	+++	+++
PI34:3	855.6	[M+Na]^+^	0	0	+	0	+	+++
PS40:1	868.7	[M+Na]^+^	0	0	++	++	++	0
PS42:8	882.6	[M+Na]^+^	0	0	0	++	+	++

+ = Detected in only one replicate.

++ = Detected in two replicates.

+++ = Detected in all three replicates.

0 = Undetectable.
