# Supplementary material for: Differential human urinary lipid profiles using various lipid-extraction protocols: MALDI-TOF and LIFT-TOF/TOF analyses
Source: Sci Rep. 2016 Sep 20;6:33756. doi: 10.1038/srep33756 (PMC5028741; doi:10.1038/srep33756)
Supplement: Supplementary Information [file srep33756-s1.pdf]

Differential human urinary lipid profiles using various lipid-extraction protocols: MALDI-TOF and LIFT-TOF/TOF analyses

*Phornpimon Tipthara and Visith Thongboonkerd**

Medical Proteomics Unit, Office for Research and Development, Faculty of Medicine Siriraj Hospital; and Center for Research in Complex Systems Science (CRCSS), Mahidol University, Bangkok, THAILAND

* E-mail: thongboonkerd@dr.com (or) vthongbo@yahoo.com

Supplementary Table S1: Details for chemical structure of lipid species identified by LIPID MAPS Structure Database.

LM_ID	Name	Systematic Name	Input *m/z*	Exact *m/z*	Formula	Adduct
Sphingomyelins						
LMSP03010003	SM(d18:1/16:0)	N-(hexadecanoyl)-sphing-4-enine-1-phosphocholine	703.7	703.5748	C39H80N2O6P	M+H
LMSP03010006	SM(d18:1/22:0)	N-(docosanoyl)-sphing-4-enine-1-phosphocholine	787.8	787.6687	C45H92N2O6P	M+H
LMSP03010008	SM(d18:1/24:0)	N-(tetracosanoyl)-sphing-4-enine-1-phosphocholine	815.8	815.7	C47H96N2O6P	M+H
Glycerophosphatidylcholines						
LMGP01010594	PC(16:0/18:2(9Z,12Z))	1-hexadecanoyl-2-(9Z,12Z-octadecadienoyl)-sn-glycero-3-phosphocholine	758.7	758.5694	C42H81NO8P	M+H
LMGP01010688	PC(16:1(9Z)/18:1(9Z))	1-(9Z-hexadecenoyl)-2-(9Z-octadecenoyl)-sn-glycero-3-phosphocholine	758.7	758.5694	C42H81NO8P	M+H
LMGP01010887	PC(18:1(9Z)/16:1(9Z))	1-(9Z-octadecenoyl)-2-(9Z-hexadecenoyl)-sn-glycero-3-phosphocholine	758.7	758.5694	C42H81NO8P	M+H
LMGP01010932	PC(18:2(9Z,12Z)/16:0)	1-(9Z,12Z-octadecadienoyl)-2-hexadecanoyl-sn-glycero-3-phosphocholine	758.7	758.5694	C42H81NO8P	M+H
LMGP01010005	PC(16:0/18:1(9Z))	1-hexadecanoyl-2-(9Z-octadecenoyl)-sn-glycero-3-phosphocholine	760.7	760.5851	C42H83NO8P	M+H
LMGP01010744	PC(18:0/16:1(9Z))	1-octadecanoyl-2-(9Z-hexadecenoyl)-sn-glycero-3-phosphocholine	760.7	760.5851	C42H83NO8P	M+H
LMGP01010884	PC(18:1(9Z)/16:0)	1-(9Z-octadecenoyl)-2-hexadecanoyl-sn-glycero-3-phosphocholine	760.7	760.5851	C42H83NO8P	M+H

LMGP01011483	PC(16:1(9Z)/18:0)	1-(9Z-hexadecenoyl)-2-octadecanoyl-glycero-3-phosphocholine	760.7	760.5851	C42H83NO8P	M+H
LMGP01010007	PC(16:0/20:4(5Z,8Z,11Z,14Z))	1-hexadecanoyl-2-(5Z,8Z,11Z,14Z-eicosatetraenoyl)-sn-glycero-3-phosphocholine	782.7	782.5694	C44H81NO8P	M+H
LMGP01010937	PC(18:2(9Z,12Z)/18:2(9Z,12Z))	1,2-di-(9Z,12Z-octadecadienoyl)-sn-glycero-3-phosphocholine	782.7	782.5694	C44H81NO8P	M+H
LMGP01011049	PC(20:4(5Z,8Z,11Z,14Z)/16:0)	1-(5Z,8Z,11Z,14Z-eicosatetraenoyl)-2-hexadecanoyl-sn-glycero-3-phosphocholine	782.7	782.5694	C44H81NO8P	M+H
LMGP01011491	PC(16:1(9Z)/20:3(8Z,11Z,14Z))	1-(9Z-hexadecenoyl)-2-(8Z,11Z,14Z-eicosatrienoyl)-glycero-3-phosphocholine	782.6	782.5694	C44H81NO8P	M+H
LMGP01011603	PC(18:1(9Z)/18:3(6Z,9Z,12Z))	1-(9Z-octadecenoyl)-2-(6Z,9Z,12Z-octadecatrienoyl)-glycero-3-phosphocholine	782.6	782.5694	C44H81NO8P	M+H
LMGP01011652	PC(18:3(6Z,9Z,12Z)/18:1(9Z))	1-(6Z,9Z,12Z-octadecatrienoyl)-2-(9Z-octadecenoyl)-glycero-3-phosphocholine	782.6	782.5694	C44H81NO8P	M+H
LMGP01011873	PC(20:3(8Z,11Z,14Z)/16:1(9Z))	1-(8Z,11Z,14Z-eicosatrienoyl)-2-(9Z-hexadecenoyl)-glycero-3-phosphocholine	782.6	782.5694	C44H81NO8P	M+H
LMGP01012136	PC(16:0/20:4(8Z,11Z,14Z,17Z))	1-hexadecanoyl-2-(8Z,11Z,14Z,17Z-eicosatetraenoyl)-sn-glycero-3-phosphocholine	782.6	782.5694	C44H81NO8P	M+H
LMGP01012138	PC(16:1(9Z)/20:3(5Z,8Z,11Z))	1-(9Z-hexadecenoyl)-2-(5Z,8Z,11Z-eicosatrienoyl)-sn-glycero-3-phosphocholine	782.6	782.5694	C44H81NO8P	M+H
LMGP01012195	PC(20:3(5Z,8Z,11Z)/16:1(9Z))	1-(5Z,8Z,11Z-eicosatrienoyl)-2-(9Z-hexadecenoyl)-sn-glycero-3-phosphocholine	782.6	782.5694	C44H81NO8P	M+H
LMGP01010005	PC(16:0/18:1(9Z))	1-hexadecanoyl-2-(9Z-octadecenoyl)-sn-glycero-3-phosphocholine	782.7	782.567	C42H82NO8PNa	M+Na
LMGP01010744	PC(18:0/16:1(9Z))	1-octadecanoyl-2-(9Z-hexadecenoyl)-sn-glycero-3-phosphocholine	782.6	782.567	C42H82NO8PNa	M+Na
LMGP01010884	PC(18:1(9Z)/16:0)	1-(9Z-octadecenoyl)-2-hexadecanoyl-sn-glycero-3-phosphocholine	782.7	782.567	C42H82NO8PNa	M+Na
LMGP01011483	PC(16:1(9Z)/18:0)	1-(9Z-hexadecenoyl)-2-octadecanoyl-glycero-3-phosphocholine	782.6	782.567	C42H82NO8PNa	M+Na
LMGP01010768	PC(18:0/18:2(9Z,12Z))	1-octadecanoyl-2-(9Z,12Z-octadecadienoyl)-sn-glycero-3-phosphocholine	786.7	786.6007	C44H85NO8P	M+H
LMGP01010890	PC(18:1(9Z)/18:1(9Z))	1,2-di-(9Z-octadecenoyl)-sn-glycero-3-phosphocholine	786.7	786.6007	C44H85NO8P	M+H
LMGP01010935	PC(18:2(9Z,12Z)/18:0)	1-(9Z,12Z-octadecadienoyl)-2-octadecanoyl-sn-glycero-3-phosphocholine	786.7	786.6007	C44H85NO8P	M+H

Glycerophosphatidylethanolamine						
LMGP02010839	PE(20:0/22:2(13Z,16Z))	1-eicosanoyl-2-(13Z,16Z-docosadienoyl)-glycero-3-phosphoethanolamine	850.7	850.6296	C47H90NO8PNa	M+Na
LMGP02010897	PE(20:2(11Z,14Z)/22:0)	1-(11Z,14Z-eicosadienoyl)-2-docosanoyl-glycero-3-phosphoethanolamine	850.7	850.6296	C47H90NO8PNa	M+Na
LMGP02011031	PE(22:0/20:2(11Z,14Z))	1-docosanoyl-2-(11Z,14Z-eicosadienoyl)-glycero-3-phosphoethanolamine	850.7	850.6296	C47H90NO8PNa	M+Na
LMGP02011090	PE(22:2(13Z,16Z)/20:0)	1-(13Z,16Z-docosadienoyl)-2-eicosanoyl-glycero-3-phosphoethanolamine	850.7	850.6296	C47H90NO8PNa	M+Na
Glycerophosphatidylglycerols						
LMGP04010351	PG(18:2(9Z,12Z)/18:3(6Z,9Z,12Z))	1-(9Z,12Z-octadecadienoyl)-2-(6Z,9Z,12Z-octadecatrienoyl)-glycero-3-phospho-(1'-sn-glycerol)	791.5	791.4834	C42H73O10PNa	M+Na
LMGP04010379	PG(18:3(6Z,9Z,12Z)/18:2(9Z,12Z))	1-(6Z,9Z,12Z-octadecatrienoyl)-2-(9Z,12Z-octadecadienoyl)-glycero-3-phospho-(1'-sn-glycerol)	791.5	791.4834	C42H73O10PNa	M+Na
LMGP04010626	PG(20:4(5Z,8Z,11Z,14Z)/16:1(9Z))	1-(5Z,8Z,11Z,14Z-eicosatetraenoyl)-2-(9Z-hexadecenoyl)-glycero-3-phospho-(1'-sn-glycerol)	791.5	791.4834	C42H73O10PNa	M+Na
LMGP04010654	PG(20:5(5Z,8Z,11Z,14Z,17Z)/16:0)	1-(5Z,8Z,11Z,14Z,17Z-eicosapentaenoyl)-2-hexadecanoyl-glycero-3-phospho-(1'-sn-glycerol)	791.5	791.4834	C42H73O10PNa	M+Na
LMGP04010899	PG(16:1(9Z)/20:4(5Z,8Z,11Z,14Z))	1-(9Z-hexadecenoyl)-2-(5Z,8Z,11Z,14Z-eicosatetraenoyl)-glycero-3-phospho-(1'-sn-glycerol)	791.5	791.4834	C42H73O10PNa	M+Na
LMGP04010903	PG(16:0/20:5(5Z,8Z,11Z,14Z,17Z))	1-hexadecanoyl-2-(5Z,8Z,11Z,14Z,17Z-eicosapentaenoyl)-glycero-3-phospho-(1'-sn-glycerol)	791.5	791.4834	C42H73O10PNa	M+Na
LMGP04010390	PG(18:3(6Z,9Z,12Z)/20:5(5Z,8Z,11Z,14Z,17Z))	1-(6Z,9Z,12Z-octadecatrienoyl)-2-(5Z,8Z,11Z,14Z,17Z-eicosapentaenoyl)-glycero-3-phospho-(1'-sn-glycerol)	813.4	813.4677	C44H71O10PNa	M+Na
LMGP04010661	PG(20:5(5Z,8Z,11Z,14Z,17Z)/18:3(6Z,9Z,12Z))	1-(5Z,8Z,11Z,14Z,17Z-eicosapentaenoyl)-2-(6Z,9Z,12Z-octadecatrienoyl)-glycero-3-phospho-(1'-sn-glycerol)	813.4	813.4677	C44H71O10PNa	M+Na
LMGP04010224	PG(16:1(9Z)/22:2(13Z,16Z))	1-(9Z-hexadecenoyl)-2-(13Z,16Z-docosadienoyl)-glycero-3-phospho-(1'-sn-glycerol)	823.7	823.546	C44H81O10PNa	M+Na
LMGP04010334	PG(18:1(9Z)/20:2(11Z,14Z))	1-(9Z-octadecenoyl)-2-(11Z,14Z-eicosadienoyl)-glycero-3-phospho-(1'-sn-glycerol)	823.7	823.546	C44H81O10PNa	M+Na

LMGP04010357	PG(18:2(9Z,12Z)/20:1(11Z))	1-(9Z,12Z-octadecadienoyl)-2-(11Z-eicosenoyl)-glycero-3-phospho-(1'-sn-glycerol)	823.7	823.546	C44H81O10PNa	M+Na
LMGP04010385	PG(18:3(6Z,9Z,12Z)/20:0)	1-(6Z,9Z,12Z-octadecatrienoyl)-2-eicosanoyl-glycero-3-phospho-(1'-sn-glycerol)	823.7	823.546	C44H81O10PNa	M+Na
LMGP04010518	PG(20:0/18:3(6Z,9Z,12Z))	1-eicosanoyl-2-(6Z,9Z,12Z-octadecatrienoyl)-glycero-3-phospho-(1'-sn-glycerol)	823.7	823.546	C44H81O10PNa	M+Na
LMGP04010541	PG(20:1(11Z)/18:2(9Z,12Z))	1-(11Z-eicosenoyl)-2-(9Z,12Z-octadecadienoyl)-glycero-3-phospho-(1'-sn-glycerol)	823.7	823.546	C44H81O10PNa	M+Na
LMGP04010570	PG(20:2(11Z,14Z)/18:1(9Z))	1-(11Z,14Z-eicosadienoyl)-2-(9Z-octadecenoyl)-glycero-3-phospho-(1'-sn-glycerol)	823.7	823.546	C44H81O10PNa	M+Na
LMGP04010600	PG(20:3(8Z,11Z,14Z)/18:0)	1-(8Z,11Z,14Z-eicosatrienoyl)-2-octadecanoyl-glycero-3 phospho-(1'-sn-glycerol)	823.7	823.546	C44H81O10PNa	M+Na
LMGP04010763	PG(22:2(13Z,16Z)/16:1(9Z))	1-(13Z,16Z-docosadienoyl)-2-(9Z-hexadecenoyl)-glycero-3-phospho-(1'-sn-glycerol)	823.7	823.546	C44H81O10PNa	M+Na
LMGP04010885	PG(18:0/20:3(8Z,11Z,14Z))	1-octadecanoyl-2-(8Z,11Z,14Z-eicosatrienoyl)-glycero-3 phospho-(1'-sn-glycerol)	823.7	823.546	C44H81O10PNa	M+Na
LMGP04010040	PG(18:0/22:6(4Z,7Z,10Z,13Z,16Z,19Z))	1-octadecanoyl-2-(4Z,7Z,10Z,13Z,16Z,19Z-docosahexaenoyl)-sn-glycero-3-phospho-(1'-sn-glycerol)	845.4	845.5303	C46H79O10PNa	M+Na
LMGP04010365	PG(18:2(9Z,12Z)/22:4(7Z,10Z,13Z,16Z))	1-(9Z,12Z-octadecadienoyl)-2-(7Z,10Z,13Z,16Z-docosatetraenoyl)-glycero-3-phospho-(1'-sn-glycerol)	845.4	845.5303	C46H79O10PNa	M+Na
LMGP04010581	PG(20:2(11Z,14Z)/20:4(5Z,8Z,11Z,14Z))	1-(11Z,14Z-eicosadienoyl)-2-(5Z,8Z,11Z,14Z-eicosatetraenoyl)-glycero-3-phospho-(1'-sn-glycerol)	845.4	845.5303	C46H79O10PNa	M+Na
LMGP04010611	PG(20:3(8Z,11Z,14Z)/20:3(8Z,11Z,14Z))	1,2-di-(8Z,11Z,14Z-eicosatrienoyl)-sn-glycero-3-phospho-(1'-sn-glycerol)	845.4	845.5303	C46H79O10PNa	M+Na
LMGP04010640	PG(20:4(5Z,8Z,11Z,14Z)/20:2(11Z,14Z))	1-(5Z,8Z,11Z,14Z-eicosatetraenoyl)-2-(11Z,14Z-eicosadienoyl)-glycero-3-phospho-(1'-sn-glycerol)	845.4	845.5303	C46H79O10PNa	M+Na
LMGP04010800	PG(22:4(7Z,10Z,13Z,16Z)/18:2(9Z,12Z))	1-(7Z,10Z,13Z,16Z-docosatetraenoyl)-2-(9Z,12Z-octadecadienoyl)-glycero-3-phospho-(1'-sn-glycerol)	845.4	845.5303	C46H79O10PNa	M+Na
LMGP04010827	PG(22:6(4Z,7Z,10Z,13Z,16Z,19Z)/18:0)	1-(4Z,7Z,10Z,13Z,16Z,19Z-docosahexaenoyl)-2-octadecanoyl-glycero-3-phospho-(1'-sn-glycerol)	845.4	845.5303	C46H79O10PNa	M+Na
LMGP04010338	PG(18:1(9Z)/22:2(13Z,16Z))	1-(9Z-octadecenoyl)-2-(13Z,16Z-docosadienoyl)-glycero-3-phospho-(1'-sn-glycerol)	851.7	851.5773	C46H85O10PNa	M+Na

LMGP04010363	PG(18:2(9Z,12Z)/22:1(11Z))	1-(9Z,12Z-octadecadienoyl)-2-(11Z-docosenoyl)-glycero-3-phospho-(1'-sn-glycerol)	851.7	851.5773	C46H85O10PNa	M+Na
LMGP04010392	PG(18:3(6Z,9Z,12Z)/22:0)	1-(6Z,9Z,12Z-octadecatrienoyl)-2-docosanoyl-glycero-3-phospho-(1'-sn-glycerol)	851.7	851.5773	C46H85O10PNa	M+Na
LMGP04010420	PG(18:3(9Z,12Z,15Z)/22:0)	1-(9Z,12Z,15Z-octadecatrienoyl)-2-docosanoyl-glycero-3-phospho-(1'-sn-glycerol)	851.7	851.5773	C46H85O10PNa	M+Na
LMGP04010522	PG(20:0/20:3(8Z,11Z,14Z))	1-eicosanoyl-2-(8Z,11Z,14Z-eicosatrienoyl)-glycero-3-phospho-(1'-sn-glycerol)	851.7	851.5773	C46H85O10PNa	M+Na
LMGP04010548	PG(20:1(11Z)/20:2(11Z,14Z))	1-(11Z-eicosenoyl)-2-(11Z,14Z-eicosadienoyl)-glycero-3-phospho-(1'-sn-glycerol)	851.7	851.5773	C46H85O10PNa	M+Na
LMGP04010578	PG(20:2(11Z,14Z)/20:1(11Z))	1-(11Z,14Z-eicosadienoyl)-2-(11Z-eicosenoyl)-glycero-3-phospho-(1'-sn-glycerol)	851.7	851.5773	C46H85O10PNa	M+Na
LMGP04010608	PG(20:3(8Z,11Z,14Z)/20:0)	1-(8Z,11Z,14Z-eicosatrienoyl)-2-eicosanoyl-glycero-3-phospho-(1'-sn-glycerol)	851.7	851.5773	C46H85O10PNa	M+Na
LMGP04010711	PG(22:0/18:3(6Z,9Z,12Z))	1-docosanoyl-2-(6Z,9Z,12Z-octadecatrienoyl)-glycero-3-phospho-(1'-sn-glycerol)	851.7	851.5773	C46H85O10PNa	M+Na
LMGP04010712	PG(22:0/18:3(9Z,12Z,15Z))	1-docosanoyl-2-(9Z,12Z,15Z-octadecatrienoyl)-glycero-3-phospho-(1'-sn-glycerol)	851.7	851.5773	C46H85O10PNa	M+Na
LMGP04010738	PG(22:1(11Z)/18:2(9Z,12Z))	1-(11Z-docosenoyl)-2-(9Z,12Z-octadecadienoyl)-glycero-3-phospho-(1'-sn-glycerol)	851.7	851.5773	C46H85O10PNa	M+Na
LMGP04010768	PG(22:2(13Z,16Z)/18:1(9Z))	1-(13Z,16Z-docosadienoyl)-2-(9Z-octadecenoyl)-glycero-3-phospho-(1'-sn-glycerol)	851.7	851.5773	C46H85O10PNa	M+Na
Glycerophosphatidylinositols						
LMGP06010938	PI(16:1(9Z)/16:1(9Z))	1,2-di-(9Z-hexadecenoyl)-sn-glycero-3-phospho-(1'-myo inositol)	829.4	829.4837	C41H75O13PNa	M+Na
LMGP06010314	PI(18:2(9Z,12Z)/16:1(9Z))	1-(9Z,12Z-octadecadienoyl)-2-(9Z-hexadecenoyl)-glycero-3-phospho-(1'-myo-inositol)	855.6	855.4994	C43H77O13PNa	M+Na
LMGP06010340	PI(18:3(6Z,9Z,12Z)/16:0)	1-(6Z,9Z,12Z-octadecatrienoyl)-2-hexadecanoyl-glycero 3-phospho-(1'-myo-inositol)	855.6	855.4994	C43H77O13PNa	M+Na
LMGP06010872	PI(16:1(9Z)/18:2(9Z,12Z))	1-(9Z-hexadecenoyl)-2-(9Z,12Z-octadecadienoyl)-glycero-3-phospho-(1'-myo-inositol)	855.6	855.4994	C43H77O13PNa	M+Na
LMGP06010877	PI(16:0/18:3(6Z,9Z,12Z))	1-hexadecanoyl-2-(6Z,9Z,12Z-octadecatrienoyl)-glycero 3-phospho-(1'-myo-inositol)	855.6	855.4994	C43H77O13PNa	M+Na
Glycerophosphatidylserines						
LMGP03010547	PS(20:1(11Z)/20:0)	1-(11Z-eicosenoyl)-2-eicosanoyl-glycero-3-phosphoserine	868.7	868.6038	C46H88NO10PNa	M+Na

LMGP03010945	PS(22:0/18:1(9Z))	1-docosanoyl-2-(9Z-octadecenoyl)-glycero-3-phosphoserine	868.7	868.6038	C46H88NO10PNa	M+Na
LMGP03010948	PS(20:0/20:1(11Z))	1-eicosanoyl-2-(11Z-eicosenoyl)-glycero-3-phosphoserine	868.7	868.6038	C46H88NO10PNa	M+Na
LMGP03010955	PS(18:1(9Z)/22:0)	1-(9Z-octadecenoyl)-2-docosanoyl-glycero-3-phosphoserine	868.7	868.6038	C46H88NO10PNa	M+Na
LMGP03010588	PS(20:2(11Z,14Z)/22:6(4Z,7Z,10Z,13Z,16Z,19Z))	1-(11Z,14Z-eicosadienoyl)-2-(4Z,7Z,10Z,13Z,16Z,19Z-docosahexaenoyl)-glycero-3-phosphoserine	882.7	882.5256	C48H78NO10PNa	M+Na
LMGP03010647	PS(20:4(5Z,8Z,11Z,14Z)/22:4(7Z,10Z,13Z,16Z))	1-(5Z,8Z,11Z,14Z-eicosatetraenoyl)-2-(7Z,10Z,13Z,16Z-docosatetraenoyl)-glycero-3-phosphoserine	882.7	882.5256	C48H78NO10PNa	M+Na
LMGP03010810	PS(22:4(7Z,10Z,13Z,16Z)/20:4(5Z,8Z,11Z,14Z))	1-(7Z,10Z,13Z,16Z-docosatetraenoyl)-2-(5Z,8Z,11Z,14Z-eicosatetraenoyl)-glycero-3-phosphoserine	882.7	882.5256	C48H78NO10PNa	M+Na
LMGP03010837	PS(22:6(4Z,7Z,10Z,13Z,16Z,19Z)/20:2(11Z,14Z))	1-(4Z,7Z,10Z,13Z,16Z,19Z-docosahexaenoyl)-2-(11Z,14Z-eicosadienoyl)-glycero-3-phosphoserine	882.7	882.5256	C48H78NO10PNa	M+Na

Differential human urinary lipid profiles using various lipid-extraction protocols: MALDI-TOF and LIFT-TOF/TOF analyses

*Phornpimon Tipthara and Visith Thongboonkerd**

Supplementary Figure S1: Inter-assay consistency of MS spectra of urinary lipid profiling using six different extraction protocols. MS spectra were acquired by using positive ionization mode in the *m/z* range of 0-1,000.

Supplementary Figure S2: Zoom-in MS spectra in *m/z* range of 675-925 to demonstrate the inter-assay consistency of urinary lipid profiling using six different extraction protocols. MS spectra were acquired by using positive ionization mode.

FigureS1

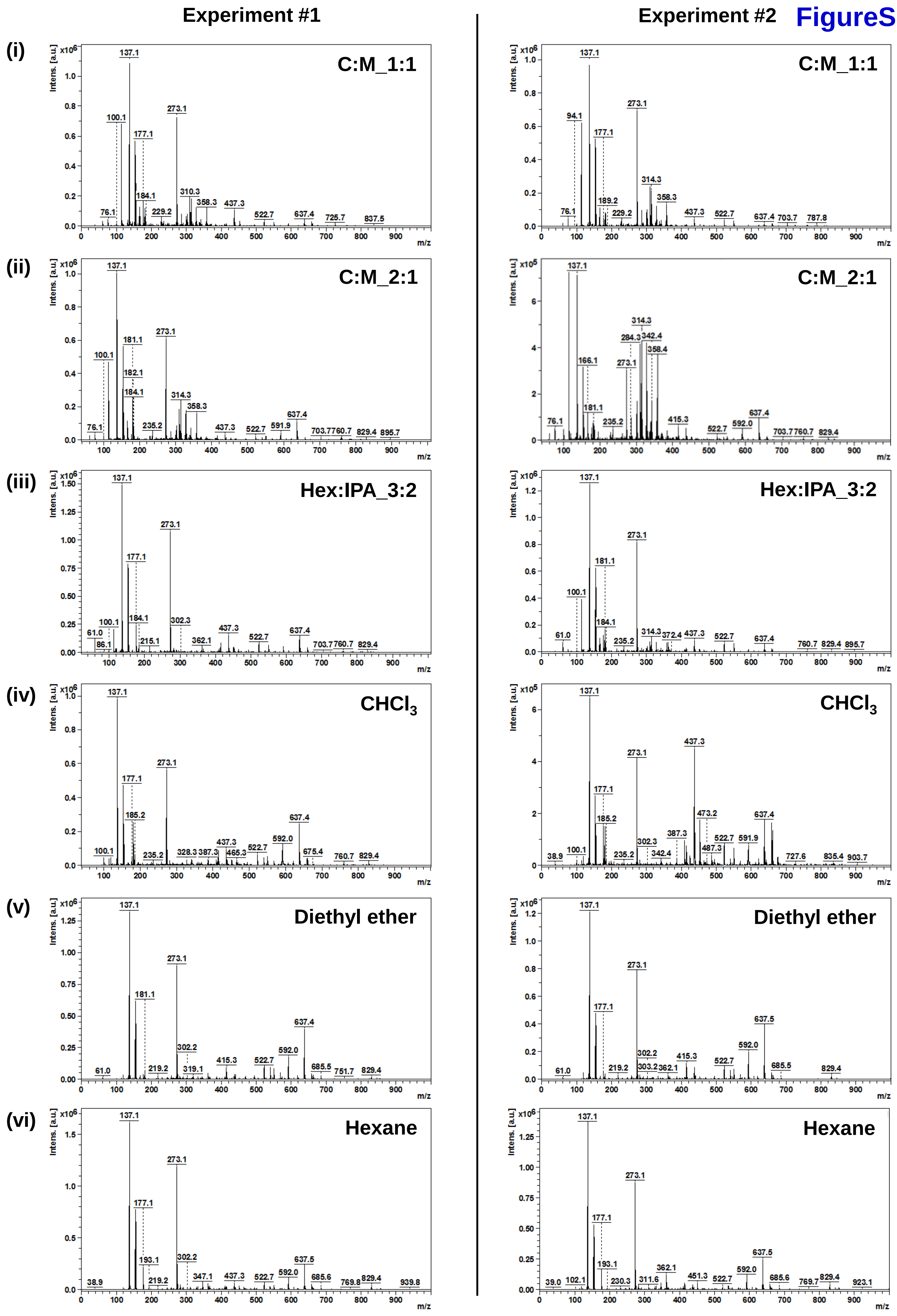

Experiment #1
Experiment #2
(i) C:M_1:1
(ii) C:M_2:1
(iii) Hex:IPA_3:2
(iv) $CHCl_3$
(v) Diethyl ether
(vi) Hexane
Intens. [a.u.]
m/z

FigureS2

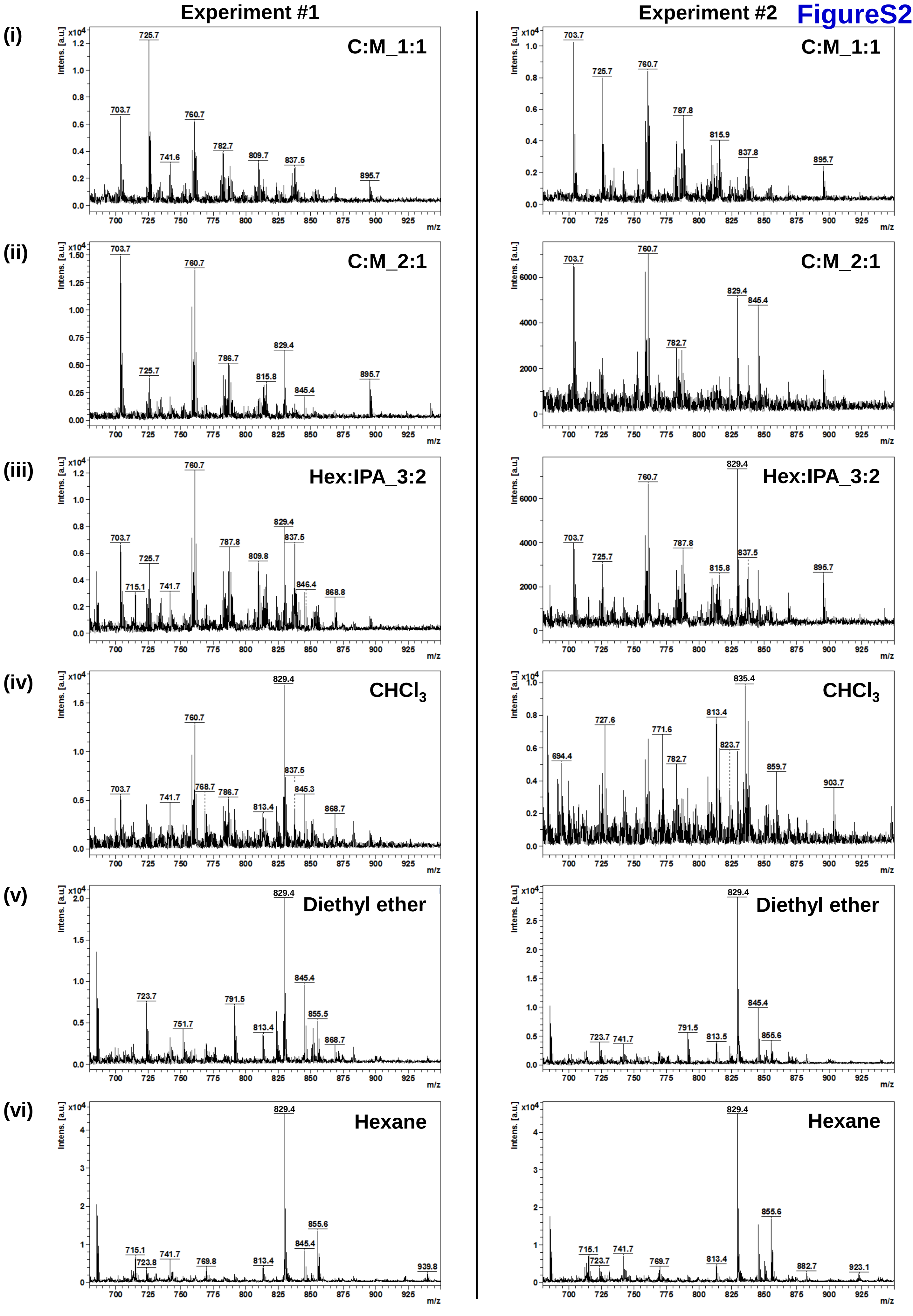
Experiment #1
Experiment #2
(i)
C:M_1:1
(ii)
C:M_2:1
(iii)
Hex:IPA_3:2
(iv)
CHCl3
(v)
Diethyl ether
(vi)
Hexane
Intens. [a.u.]
m/z
